# Supplementary material for: A chromosome-level, haplotype-resolved genome assembly and annotation for the Eurasian minnow (Leuciscidae: Phoxinus phoxinus) provide evidence of haplotype diversity
Source: Gigascience. 2025 Jan 29;14:giae116. doi: 10.1093/gigascience/giae116 (PMC11775470; doi:10.1093/gigascience/giae116)
Supplement: giae116_Supplemental_Figures_and_Tables [file giae116_supplemental_figures_and_tables.zip › Figure_S5_Supplementary Material.pdf]

A.

B.

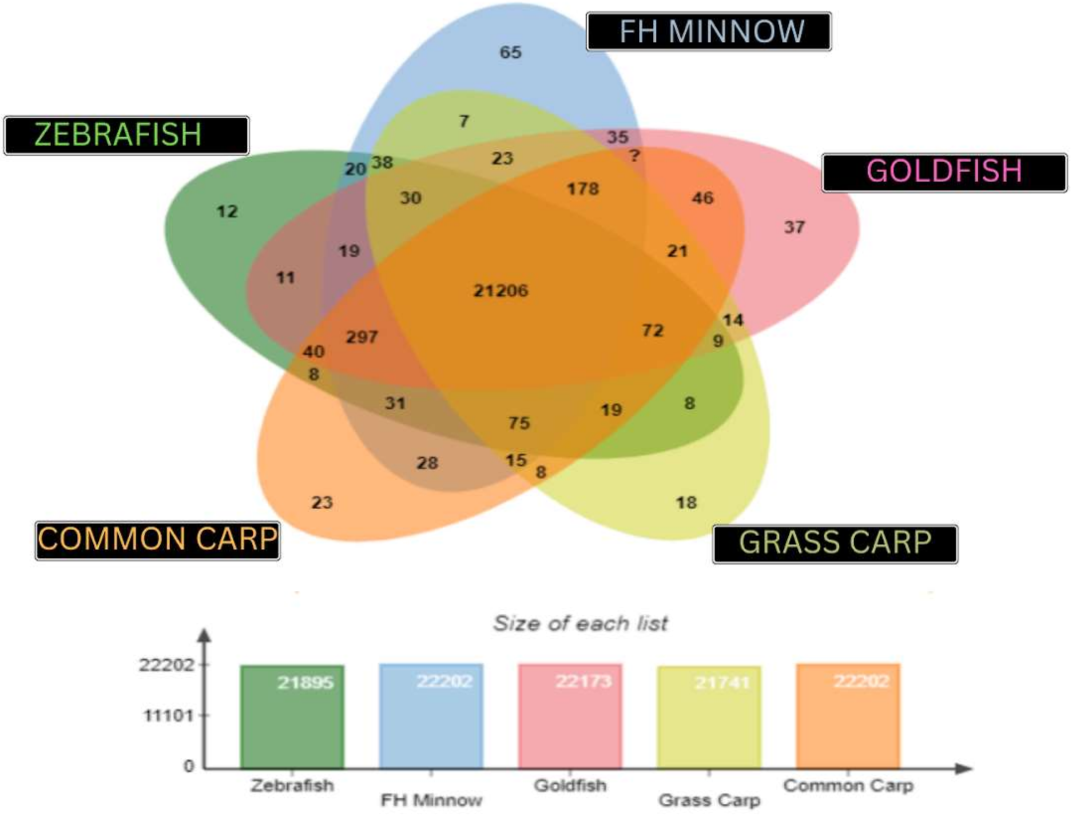


**Figure S5: Shared homologs between *Phoxinus phoxinus* and zebrafish, fathead minnow, goldfish, grass carp and common carp.**

A. Venn diagram of shared homologs between multiple fish species and the *Phoxinus phoxinus*.
B. Total number of homologs shared by each species with the Phoxinus phoxinus.
