# Supplementary material for: A chromosome-level, haplotype-resolved genome assembly and annotation for the Eurasian minnow (Leuciscidae: Phoxinus phoxinus) provide evidence of haplotype diversity
Source: Gigascience. 2025 Jan 29;14:giae116. doi: 10.1093/gigascience/giae116 (PMC11775470; doi:10.1093/gigascience/giae116)
Supplement: giae116_Supplemental_Figures_and_Tables [file giae116_supplemental_figures_and_tables.zip › Figure_S6_Supplementary Material.pdf]

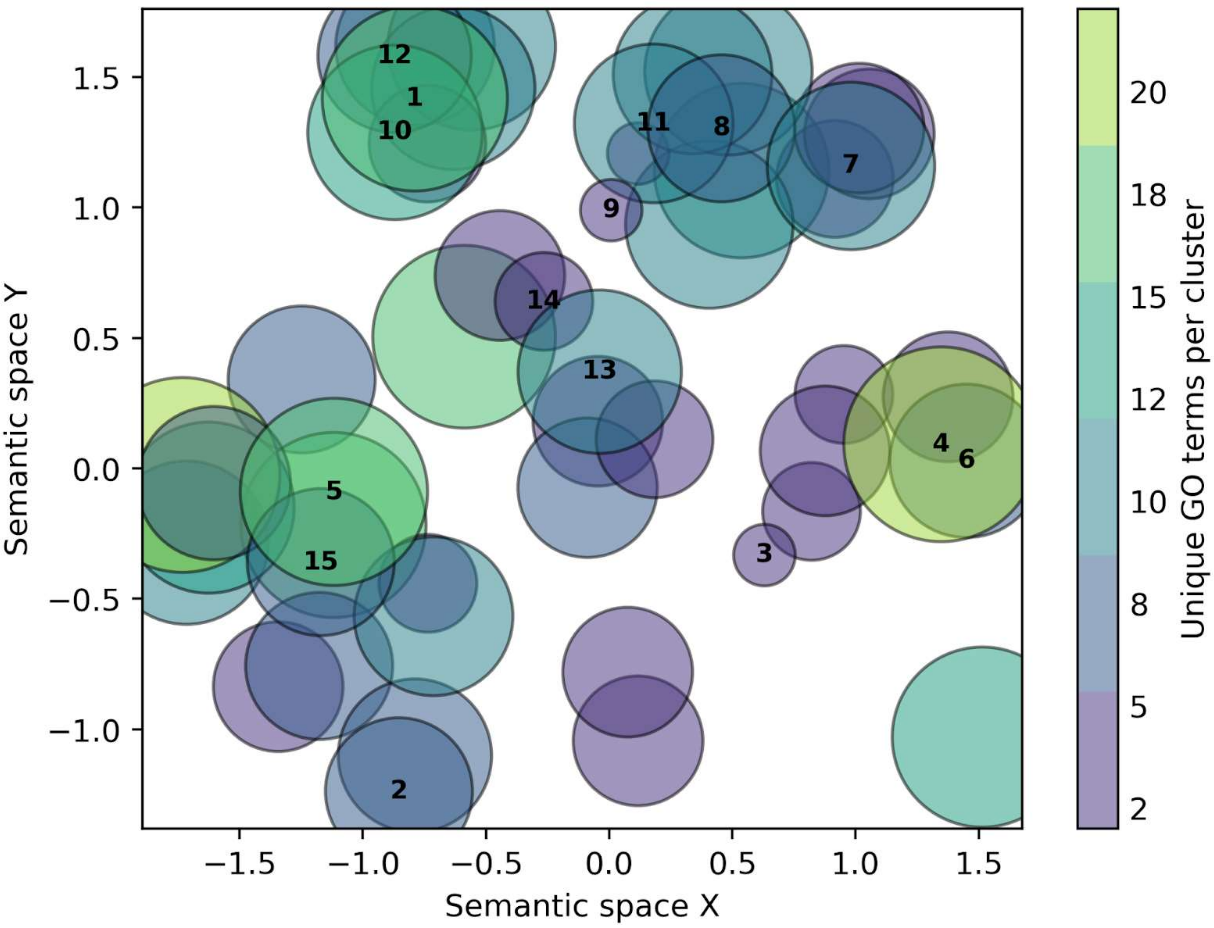

1. cerebellum vasculature development
2. netrin-activated signaling pathway
3. dephosphorylation
4. protein processing
5. regulation of calcium ion-dependent exocytosis
6. peptidyl-serine phosphorylation
7. transmission of nerve impulse
8. calcium-ion regulated exocytosis
9. lipid localization
10. neural crest cell differentiation
11. phospholipid transport
12. cell morphogenesis involved in neuron differentiat...
13. axon extension involved in axon guidance
14. developmental pigmentation
15. regulation of developmental growth

Figure S6: Gene ontology of genes in Insertion/Deletions
