# Supplementary material for: A chromosome-level, haplotype-resolved genome assembly and annotation for the Eurasian minnow (Leuciscidae: Phoxinus phoxinus) provide evidence of haplotype diversity
Source: Gigascience. 2025 Jan 29;14:giae116. doi: 10.1093/gigascience/giae116 (PMC11775470; doi:10.1093/gigascience/giae116)
Supplement: giae116_Supplemental_Figures_and_Tables [file giae116_supplemental_figures_and_tables.zip › Tables_S3_S4_Supplementary Material.pdf]

Table S3: Statistics of repeat sequences annotated in Haplotype 1

Type	Number of elements	Length occupied(bp)	% of genome
SINEs	20,701	9,685,118	1.03
LINEs	95,968	39,861,338	4.24
LTR	88,887	58,530,202	6.23
DNA Transposons	986,164	199,681,960	21.25
Simple repeats	357,271	25,011,696	2.66
Low complexity	30,066	1,632,335	0.17
Small RNA	15,964	6,297,372	0.47
Unclassified	678,241	132,275,811	14.07
Rolling-circles	28,488	19,457,434	2.07
Total Repeats Masked		506,150,215	53.86

Table S4: Statistics of repeat sequences annotated in Haplotype 2

Type	Number of elements	Length occupied(bp)	% of genome
SINEs	20,038	10,138,453	1.09
LINEs	93,623	38,556,326	4.15
LTR	87,308	57,276,336	6.16
DNA Transposons	977,301	196,769,671	21.17
Simple repeats	350,147	24,557,902	2.64
Low complexity	29,831	1,637,165	0.18
Small RNA	15,155	6,228,729	0.67
Unclassified	668,657	130,249,015	14.01
Rolling-circles	27700	20,074,520	2.16
Total Repeats Masked		499,170,338	53.71
