# Supplementary material for: A chromosome-level, haplotype-resolved genome assembly and annotation for the Eurasian minnow (Leuciscidae: Phoxinus phoxinus) provide evidence of haplotype diversity
Source: Gigascience. 2025 Jan 29;14:giae116. doi: 10.1093/gigascience/giae116 (PMC11775470; doi:10.1093/gigascience/giae116)
Supplement: giae116_Supplemental_Figures_and_Tables [file giae116_supplemental_figures_and_tables.zip › Table_S2_Supplementary Material.pdf]

Table S2: Summary statistics of the 25 largest chromosomes of both haplomes

Chromosome	Length Hap1	Length Hap2	N_counts Hap1	N_counts Hap2	GC_counts Hap1	GC_counts Hap2
chr1	54,986,461	54,993,545	6080	5480	21,640,651	21,621,225
chr2	48,474,465	52,476,322	4080	4000	19,024,086	20,630,447
chr3	47,672,159	47,342,291	6440	2600	18,449,742	18,281,120
chr4	45,191,066	42,481,172	4240	5800	17,541,055	16,516,799
chr5	44,890,755	40,393,998	3320	3720	17,622,671	15,727,182
chr6	44,260,849	44,933,711	6160	5000	17,588,082	17,886,922
chr7	40,134,292	42,607,789	4400	4680	15,662,236	16,632,902
chr8	39,332,310	38,661,445	4000	4200	15,514,313	15,225,733
chr9	39,047,206	31,210,743	2600	1880	15,680,757	12,369,284
chr10	37,018,565	37,176,178	4600	3400	14,417,725	14,477,234
chr11	36,381,605	37,099,300	5000	6000	14,084,356	14,391,568
chr12	36,320,318	36,599,732	4520	3080	14,106,636	14,221,779
chr13	36,012,166	35,414,644	4480	3600	140,13,961	13,767,350
chr14	35,931,519	33,969,163	4000	1800	14,052,979	13,294,563
chr15	35,750,743	35,292,775	4400	3200	13,924,384	13,776,540
chr16	35,231,784	35,722,923	2000	3000	13,763,990	13,977,112
chr17	35,024,062	34,260,779	3600	3000	13,723,061	13,378,019
chr18	32,836,095	32,014,133	3000	3000	12,891,976	12,568,077
chr19	32,517,511	32,398,972	3080	2800	12,718,626	12,705,184
chr20	32,504,701	33,217,526	3600	3400	12,703,219	12,964,862
chr21	32,232,413	32,235,896	3600	3880	12,521622	12,566,351
chr22	30,694,596	29,484,087	4040	1880	11,995,137	11,529,814
chr23	29,429,680	30,284,920	3800	2640	11,489,442	11,850,020
chr24	27,735,089	27,536,296	2600	3400	10,767,244	10,669,027
chr25	26,155,947	26,933,469	2720	2720	10,308,350	10,652,242
