# Supplementary material for: A chromosome-level, haplotype-resolved genome assembly and annotation for the Eurasian minnow (Leuciscidae: Phoxinus phoxinus) provide evidence of haplotype diversity
Source: Gigascience. 2025 Jan 29;14:giae116. doi: 10.1093/gigascience/giae116 (PMC11775470; doi:10.1093/gigascience/giae116)
Supplement: giae116_Supplemental_Figures_and_Tables [file giae116_supplemental_figures_and_tables.zip › Table_S5_Supplementary Material.pdf]

Table S5: Summary mapping statistics of RNA sequence data used for protein annotation in the *Phoxinus phoxinus* genome

Sample	Read Length	Total Sequences	Reads Mapped Hap1	Reads Mapped Hap2	Mapping % Hap1	Mapping % Hap2
Brain	148	14,569,393,866	14,470,079,592	14,475,502,985	99.32	99.36
Gill	148	14,866,778,876	14,757,045,591	14,749,480,068	99.26	99.21
Gonad	148	15,792,931,193	15,650,843,930	15,602,416,417	99.10	98.79
liver	148	15,180,023,298	15,036,318,160	14,793,448,652	99.05	97.45
Muscle	148	14,416,254,932	14,279,684,294	13,979,419,332	99.05	96.97
Skin	148	13,858,527,681	13,753,002,576	13,853,592,946	99.24	99.96
Spleen	148	14,035,619,158	13,903,999,665	13,832,603,441	99.06	98.55
