# Supplementary figures and images for: A chromosome-level, haplotype-resolved genome assembly and annotation for the Eurasian minnow (Leuciscidae: Phoxinus phoxinus) provide evidence of haplotype diversity
Source: Gigascience. 2025 Jan 29;14:giae116. doi: 10.1093/gigascience/giae116 (PMC11775470; doi:10.1093/gigascience/giae116)

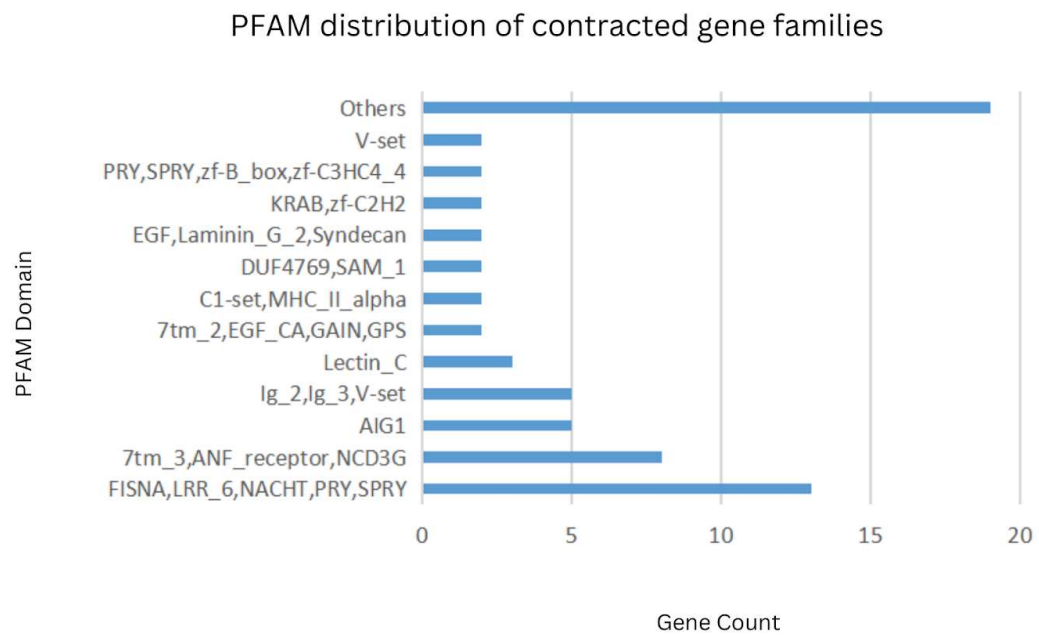

**Figure S10: Pfam domain distribution of contracted genes**

Supplement: giae116_Supplemental_Figures_and_Tables [file giae116_supplemental_figures_and_tables.zip › Figure_S10_Supplementary Material.pdf]

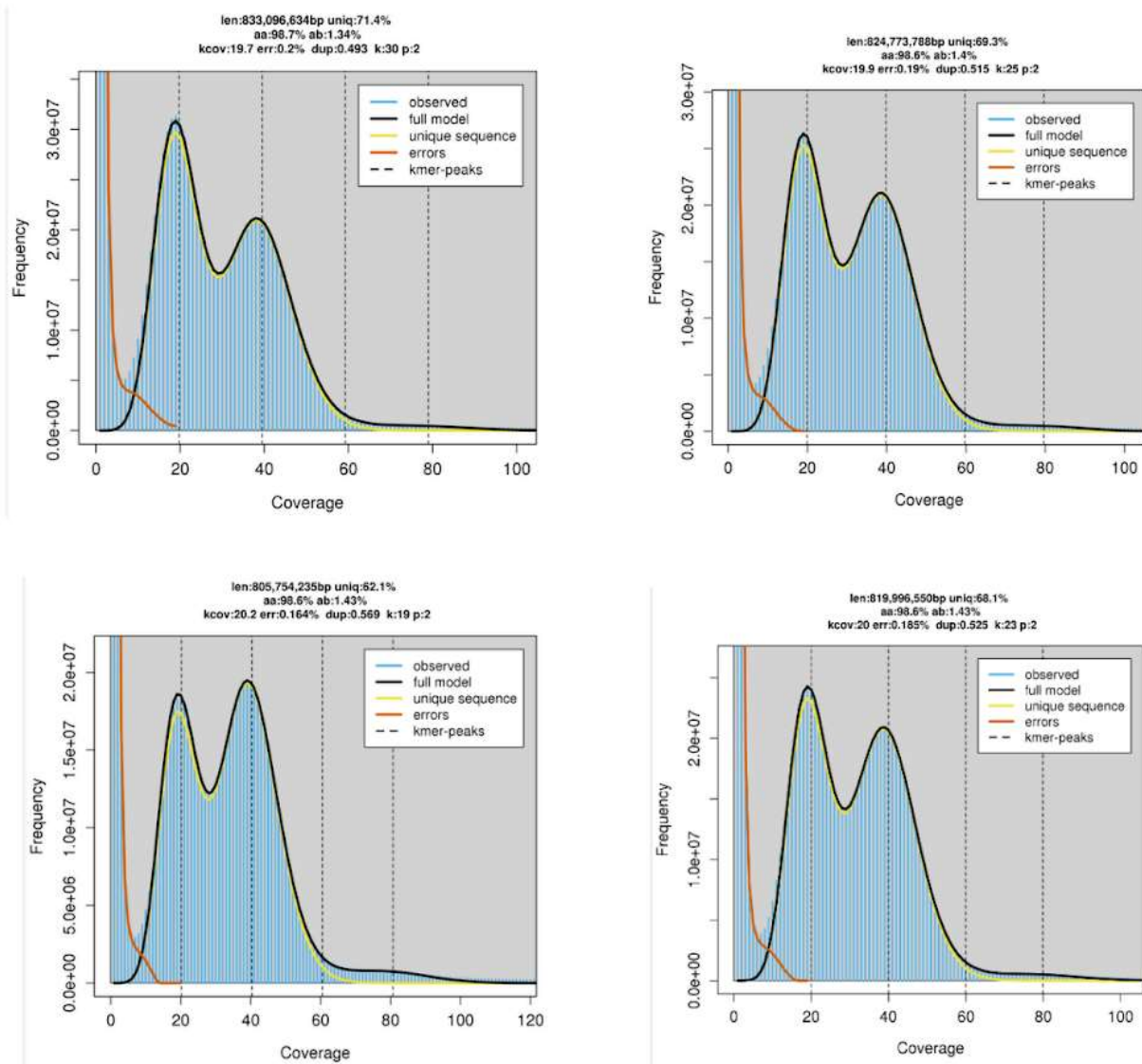

**Figure S3: GenomeScope plots of 19, 23, 25 and 30 mer analysis**

Supplement: giae116_Supplemental_Figures_and_Tables [file giae116_supplemental_figures_and_tables.zip › Figure_S3_Supplementary Material.pdf]

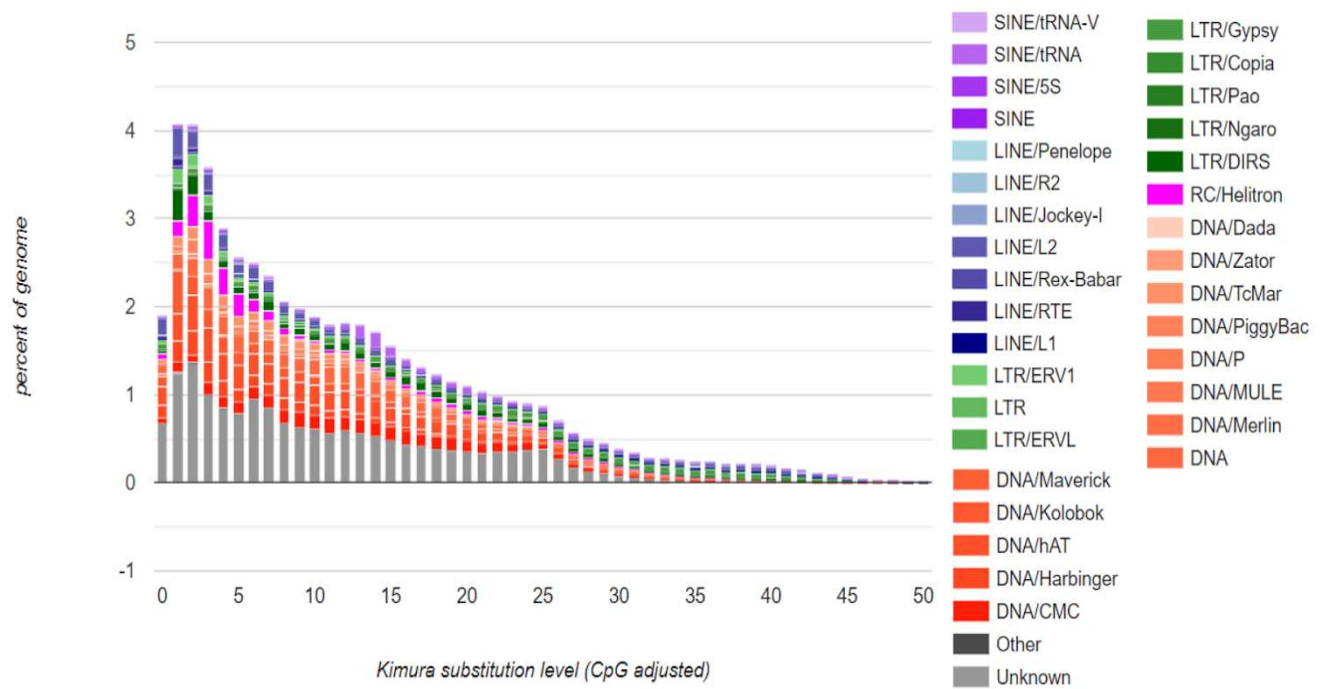

**Figure S4: Repeat Landscape of annotated repeats in Haplotype 2**

Supplement: giae116_Supplemental_Figures_and_Tables [file giae116_supplemental_figures_and_tables.zip › Figure_S4_Supplementary Material.pdf]
