# Supplementary material for: A chromosome-level, haplotype-resolved genome assembly and annotation for the Eurasian minnow (Leuciscidae: Phoxinus phoxinus) provide evidence of haplotype diversity
Source: Gigascience. 2025 Jan 29;14:giae116. doi: 10.1093/gigascience/giae116 (PMC11775470; doi:10.1093/gigascience/giae116)
Supplement: giae116_Reviewer_3_Report_Original_Submission — Jitendra Narayan -- 11/9/2024 [file giae116_reviewer_3_report_original_submission.pdf]

Reviewer Report

Title: A chromosome-level, haplotype-resolved genome assembly and annotation for the Eurasian minnow (Leuciscidae: Phoxinus phoxinus) provide evidence of haplotype diversity

Version: Original Submission **Date:** 11/9/2024

Reviewer name: Jitendra Narayan

Reviewer Comments to Author:

The authors present a detailed investigation into the genomic architecture of the Eurasian minnow (Phoxinus phoxinus), contributing substantially to our understanding of the species' genetic diversity, structural variation, and evolutionary adaptations. Employing a combination of high-fidelity long-read sequencing and Hi-C chromatin conformation capture, the authors constructed a haplotype-phased, chromosome-level genome assembly (2n = 50) with remarkable accuracy and completeness. The resultant assembly spans haploid genome sizes of 940 Mb and 929 Mb for each haplome, achieving scaffold N50 values of 36.4 Mb and 36.6 Mb, respectively. High BUSCO scores (96.9% and 97.2%) underscore the assembly's robustness, establishing a solid framework for subsequent genetic and evolutionary analyses.

The study's findings provide significant insights into P. phoxinus's genomic landscape, with a heterozygosity rate of 1.43% and a high repetitive content (~54%) dominated by DNA transposons, which are known to facilitate genomic rearrangements and adaptive evolution. The assembly further reveals extensive structural variation, including insertions, deletions, inversions, and translocations. Comprehensive genome annotation identified 30,980 mRNAs and 23,497 protein-coding genes, facilitating a comparative gene family analysis with ten teleost species. Results highlight a reliance on histone-mediated immune defenses over NLR-based pathways, suggesting adaptive immune strategies unique to P. phoxinus.

This high-quality, phased genome assembly not only serves as a crucial resource for resolving taxonomic complexities within Phoxinus but also underscores the utility of phased assemblies for elucidating genetic diversity in heterozygous species. The authors deliver a methodologically rigorous and data-rich resource that will be instrumental for future research in teleost genomics.

Methods

Are the methods appropriate to the aims of the study, are they well described, and are necessary controls included? Choose an item.

Conclusions

Are the conclusions adequately supported by the data shown? Choose an item.

Reporting Standards

Does the manuscript adhere to the journal's guidelines on minimum standards of reporting? Choose an item.

Choose an item.

Statistics

Are you able to assess all statistics in the manuscript, including the appropriateness of statistical tests used? Choose an item.

Quality of Written English

Please indicate the quality of language in the manuscript: Choose an item.

Declaration of Competing Interests

Please complete a declaration of competing interests, considering the following questions:

- Have you in the past five years received reimbursements, fees, funding, or salary from an organisation that may in any way gain or lose financially from the publication of this manuscript, either now or in the future?
- Do you hold any stocks or shares in an organisation that may in any way gain or lose financially from the publication of this manuscript, either now or in the future?
- Do you hold or are you currently applying for any patents relating to the content of the manuscript?
- Have you received reimbursements, fees, funding, or salary from an organization that holds or has applied for patents relating to the content of the manuscript?
- Do you have any other financial competing interests?
- Do you have any non-financial competing interests in relation to this paper?

If you can answer no to all of the above, write 'I declare that I have no competing interests' below. If your reply is yes to any, please give details below.

I declare that I have no competing interests.

I agree to the open peer review policy of the journal. I understand that my name will be included on my report to the authors and, if the manuscript is accepted for publication, my named report including any attachments I upload will be posted on the website along with the authors' responses. I agree for my report to be made available under an Open Access Creative Commons CC-BY license (http://creativecommons.org/licenses/by/4.0/). I understand that any comments which I do not wish to be included in my named report can be included as confidential comments to the editors, which will not be published.

Choose an item.

To further support our reviewers, we have joined with Publons, where you can gain additional credit to further highlight your hard work (see: https://publons.com/journal/530/gigascience). On publication of this paper, your review will be automatically added to Publons, you can then choose whether or not to claim your Publons credit. I understand this statement.

Yes Choose an item.
